# Candidate gene studies of diabetic retinopathy in human

**DOI:** 10.1007/s11033-016-4075-y

**Published:** 2016-10-11

**Authors:** Petra Priščáková, Gabriel Minárik, Vanda Repiská

**Affiliations:** 1 Faculty of Medicine, Institute of Medical Biology, Genetics and Clinical Genetics, University Hospital Bratislava, Comenius University in Bratislava, Sasinkova 4, 81108 Bratislava, Slovakia; 2 Medirex Group Academy n.o., Galvaniho 17/C, 82016 Bratislava, Slovakia

**Keywords:** Diabetic retinopathy, DNA variants, Sequencing, *Diabetes mellitus*, Genetic studies

## Abstract

Diabetic retinopathy (DR) is a multifactorial disease with complex pathophysiology. It is the main cause of blindness among the people in productive age. The purpose of this literature review is to highlight recent achievements in the genetics of diabetic retinopathy with particular focus on candidate gene studies. We summarized most of the available published data about candidate genes for diabetic retinopathy with the goal to identify main genetic aspects. We conclude that genetic studies reported contradictory findings and no genetic variants meet criteria of a diagnostic marker, or significantly elucidate the root of DR development. Based on these findings it is important to continue with the research in the field of DR genetics, mainly due to the fact that currently new possibilities and approaches associated with utilization of next-generation sequencing are available.

## Introduction

Diabetes mellitus (DM) is one of the most significant health problems worldwide. It is a metabolic disorder in which elevated blood sugar levels are present as a result of the inability to produce a sufficient amount of insulin (type 1) or because of cellular insulin resistance (type 2). Both types of diabetes are associated with hyperglycaemia, oxidative stress, inflammation and macrovascular (coronary artery disease, atherosclerosis, hypertension and stroke) and microvascular complications such as retinopathy, nephropathy and neuropathy [1].

The number of patients with diabetes mellitus is rapidly increasing every year. Global mortality resulting from diabetes in adults was estimated to be 1.5 million deaths in 2012 (World Health Organization). It is estimated that there will be 418 million patients with impaired glucose tolerance and 380 million patients with T2DM by 2025 [2].

**Fig. 1:**
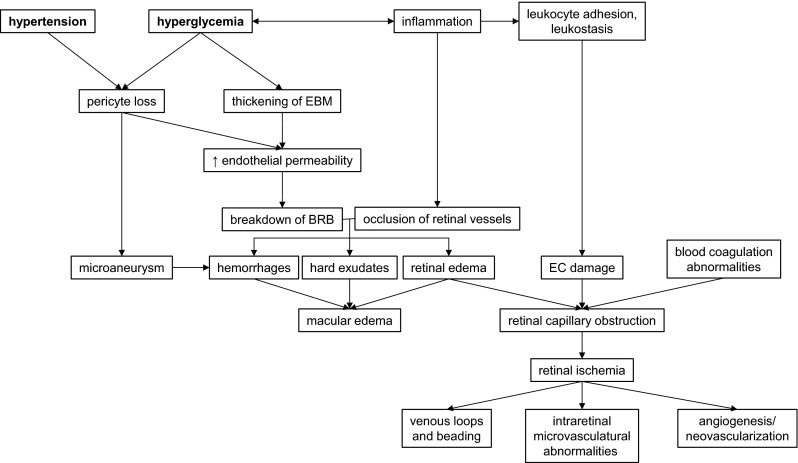
Symptoms and pathological processies typical for diabetic retinopathy leading to vision lost. *EBM* endothelial basal membrane, *BRB* blood retinal barrier, *EC* endothelial cell

Diabetic retinopathy (DR) is a leading cause of visual impairment in patients at productive age. These alarming numbers highlight the necessity of optimization of diagnostic methods that will allow early identification of diabetic patients with significantly elevated risk of DR development that will help start optimal prevention and intervention. DR has an overall prevalence of 22–37 % in individuals with known diabetes. It leads to damage of the retina microvasculature as a result of prolonged exposure to metabolic changes induced by diabetes. If left untreated, it may lead to blindness on account of continuous blood leakage due to the loss of retinal pericytes and fenestration [1]. DR is classified into two categories based on severity, namely less-severe nonproliferative diabetic retinopathy (NPDR) and severe proliferative diabetic retinopathy (PDR). The key changes of the retina in NPDR, as a result of hypoxia and venous bleading, are microaneurysms, vascular leakage, hard exudates, intraretinal microvascular abnormalities and cotton wool spots (Fig. 1). Retinal neovascularization induced by ischemia is the main characteristic of PDR [3].

**Fig. 2:**
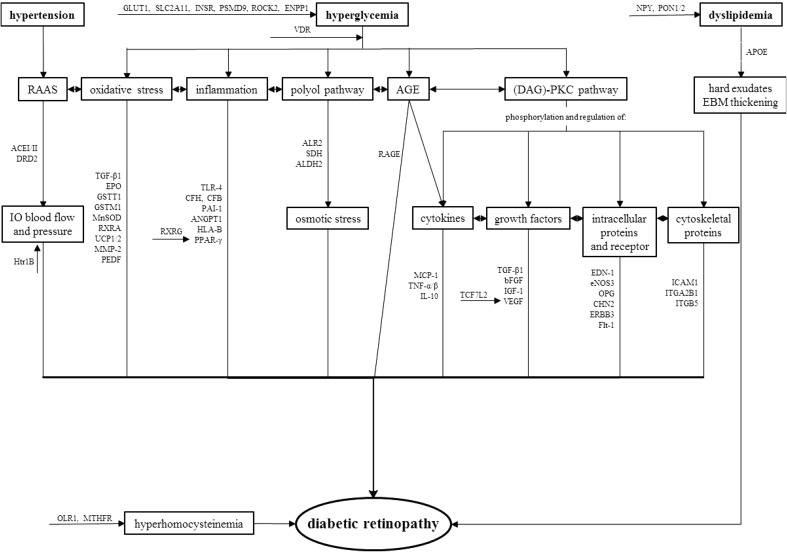
Putative roles of genes identified by candidate genes studies in pathophysiological processies during diabetic retinopathy. *RAAS* renin–angiotensin–aldosterone system, *AGE* advanced glycation end-product, *IO* intraoccular, *EBM* endothelial basal membrane, *BRB* blood retinal barrier

The aetiology of this complex disease remains unclear and poorly understood. It is associated with both environmental and genetic factors. The possibility of developing and progression of DR is closely related to the duration of DM [1]. Almost all patients with T1DM and >60 % of patients with T2DM are anticipated to have some type of retinopathy within the first 10 years of diabetes being diagnosed. The Diabetes Control and Complications Trial (DCCT) and United Kingdom Prospective Diabetes Study (UKPDS) clinical trials have also confirmed significant association between chronic hyperglycaemia and development and progression of DR, however, the fundamentals of how hyperglycaemia causes microvascular changes in the retina has not been fully elucidated [4]. Involvement of several biochemical pathways which can elucidate the role of hyperglycaemia in DR pathophysiology has been proposed, including activation of diacyl-glycerol **(**DAG)-PKC pathway, accelerated formation of advanced glycation endproducts (AGE), increased polyol pathway flux, increased expression of growth factors (VEGF, IGF-1), haemodynamic changes, renin-angiotensine-aldosterone system (RAAS), leukostasis, subclinical inflammation, and oxidative stress that leads to increased expression of several proinflammatory genes (NF-κB, TGF-β, NOX4, Nrf2, etc.). Other risk contributors for DR development are dyslipidemia [5] and possibly blood pressure, but studies are contradictory about these risk modifiers [4, 6]. Pathways contributing to DR pathologies alongside with relevant genes are summarized in Fig. 2. In summary, increased vascular permeability, haemostatic abnormalities, endothelial dysfunction, increased tissue ischemia, angiogenesis and neovascularization is typical for overall DR pathophysiology [1].

## Genetic aspects of the diabetic retinopathy

The above mentioned risk factors are not solely responsible for susceptibility to DR. Clinical studies have revealed considerable variations in the retinopathy onset and severity that cannot be fully explained by known risk factors such as the duration of diabetes, the level of glycemic control, or concomitant vascular disease [7]. For instance, some people might have DR even when they have good glycemic control and duration of DM is short. In contrast, other patients have poor glycemic control and prolonged duration of DM and yet may not develop DR. The probability of DR development also depends on the ethnicity; the Hispanics, the individuals of African descent and the Asians are more susceptible to DR [8, 9]. A study from 2008 reported retinopathy symptoms, including retinal microaneurysms, in nondiabetic patients with an optimal glucose level (glycosylated haemoglobin levels <5.0 %) [10] and these microvascular changes were indistinguishable from lesions in diabetic DR. Therefore there is evidence that additional risk factors and genetic predispositions have a part in the development and progression of DR and that these factors are independent of DM.

This is a solid confirmation of genetic contribution to the development and progression of DR. Over the past several years, progress has been made in identifying some of the susceptibility loci associated with DR through twin studies, family studies, candidate gene studies, linkage studies and small-scale GWAS (genome-wide association study). Twin and family studies have demonstrated that risk of DR emergence is three times higher for patients with a family history of DR than in patients without it for both T1DM and T2DM. Concordance is dramatically higher among monozygotic twins when compared to dizygotic twins [11]. One of the first twin studies has reported DR concordance of 68 % in T1DM and 95 % in T2DM [12]. Heritability score increases with the severity of DR, and has been estimated to be 18 and 52 % for DR and PDR, respectively [13].

## Candidate genes studies

Our knowledge of pathophysiology of DR allows us to propose possible candidate genes, which could play a role in the development and progression of DR. Candidate gene studies compare the frequency of a particular genetic variant in subjects with or without DR. This approach has revealed several genes with a possible key role in DR. These genes are part of different physiological and pathophysiological processes in organism, often associated with inflammation, such as RAAS (renin–angiotensine–aldosterone system), glucose induced pathways, remodeling of extracellular matrix (ECM), vascular endothelial dysfunction, and angiogenesis. It has been proposed that a great number of factors and genes with modest effect, as a part of different biochemical pathways, invoke pathological processes leading to DR.

## Polyol pathway and its role in DR

Polyol pathway represents the main metabolic link between hyperglycaemia and damages caused by DM. Aldose reductase (ALR2) is the essentialenzyme in the pathway. ALR2 converts glucose to sorbitol in an NADPH-dependent reaction. During the hyperglycaemia sorbitol accumulates in cells and induces osmotic stress and cellular damage. The above mentioned process leads to the destruction of retinal cells, microaneurysms, thickening of the basement membrane and loss of pericytes in animal models, which are also typical symptoms of DR in humans [14]. Three DR-associated ALR2 polymorphisms have been identified in different populations (see Table 1). The first polymorphism located at the 5′ end of the gene, the Z-2 allele of the (CA)_n_ microsatellite located at the 5′ end of the gene increases risk of DR. In contrast, Z + 2 and Z alleles show protective effect against DR [15]. Another polymorphism, rs759853, has shown association with DR where T allele confers protection against DR in T1DM [15, 16]. However, the use of AKR (aldo–keto reductase) inhibitors did not confirm expected results in clinical trials and they could not prevent progression of the disease [17]. But these clinical trials disregarded the genetic variants in the *AKR2* gene which could have had negative impact on the function of AKR inhibitors. Another enzyme in the polyol pathway, sorbitol dehydrogenase (SDH) converts sorbitol into fructose in NAD^+^-dependent reaction. Amano et al. found that *SDH* overexpression potentiated glucose toxicity to cultured retinal pericytes, thus leading to acceleration of pericyte loss, a typical trait of DR [18]. Polymorphisms rs2055858 and rs3759890 were identified in Polish, Japanese and Caucasian-Brazilian population and it is possible that they could affect the promoter activity of the *SDH* gene and have role in onset of DR [18, 19].

**Table 1:** Summary of genes identified by candidate genes studies with possible role in pathophysiology of DR

Gene symbol	Gene name	Function/cellular role	Polymorphism	Ch.	Type of DM	Population	Comments	Ref.
AKR1B1/ALR2	Aldose reductase gene	Polyol pathway—conversion of glucose to sorbitol	rs35839483 [(CA)n dinucleotide repeats]	7	1 and 2	Chinese, Japanese, Indians, Chileans, Brazilians	z-2 microsatellite confers risk in all DR, z2 microsatellite against all DR	[20–27]
			rs759853 (c. C-106T)	7	2	Euro-Brazilian, Mainland Chinese, Han Chinese, Japanese	T allele protective against DR but according to some studies it is weak association	[25, 26, 28–30]
			rs9640883	7	2	Australian	Association with onset of diabetes	[25]
SDH	Sorbitol dehydrogenase	Polyol pathway—conversion of sorbitol to fructose	rs2055858 (c. C-1214G)	15	2	Poland	Weak associations; polymorphism possibly affect promoter activity	[18, 19]
			rs3759890 (c. G-888C)	15	2	Japan, Poland, Caucasian-Brazilians	Inconsistent finding, polymorphism possibly affect promoter activity	[18, 31]
ALDH2	Mitochondrial aldehyde dehydrogenase 2	Polyol pathway—transformation from acetaldehyde to acetic acid, prevents creation of AGE	ALDH2*2	12	2	Japanese	Associated with protective effect against DR	[32]
VEGF	Vascular endothelial growth factor	Stimulation of angiogenesis and vasculogenesis	rs2010963 (c. C-634G)	6	2	Japanese, Indian, Caucasian	C allele confers risk for NPDR in T2DM	[33–41]
			(c. C-460T)	6	1 and 2	Caucasian	Possible association with DR	[42, 43]
			rs25648	6	2	Multi-ethnic	T allele increase risk of DR but finding inconsistent	[33, 34, 37]
			rs1570360 (c. A-116G)	6	2	Multi-ethnic	Inconsistent finding	[33, 34, 42]
			rs3095039	6	2	Multi-ethnic	T allele increase risk of DR but finding inconsistent	[33–36]
			rs35569394	6	1 and 2	Multi-ethnic	(− 2549) DEL increases risk but finding inconsistent	[33]
			rs699947(c. A-2578C)	6	2	Multi-ethnic	A allele increases risk but finding inconsistent	[33, 36, 40, 43–46]
			rs13207351 (c. A-152G)	6	1 and 2	Caucasian	Associated with PDR in some of the studies	[34, 42, 46]
			rs735286 (c. C4618T)	6	1 and 2	Caucasian	Haplotype-tagged SNP associated with severity of DR	[42]
			rs2146323 (c. C5092A)	6	1 and 2	Caucasian	Haplotype-tagged SNP associated with severity of DR, associated with early progression of DR	[42, 44, 47]
			rs833061 (c. C-1498T)	6	2	Chinese	Inconsistent finding and weak association	[34, 37, 39, 46]
			rs3025021	6	2	Chinese	Inconclusive	[33, 46]
			rs10434	6	1 and 21 and 2	Caucasian	G allele associated with blinding DR	[33]
			rs833068	6	1 and 2	Caucasian	G allele confers risk in DR	[33]
			rs833070	6	1	Japanese	Associated with early progression of DR but weak association	[44]
			rs3025039 (c. C+936T)	6	2	Caucasian	T allele increases risk	[48]
bFGF/FGF2	Basic fibroblast growth factor/fibroblast growth factor 2	Stimulation of angiogenesis and tissue repair	rs41456044	4	2	Multi-ethnic	A allele increases risk but weak associations	[15]
			rs308395	4	2	Multi-ethnic	G allele increases risk but finding inconsistent	[15]
			c. C-754G	4	2	Slovak	C allele increases level of bFGF	[49]
			c. T − 553 A c. T−834A	4	2	Caucasian	AT genotype could be risk factor for PDR during T2DM	[50]
IGF-1	Insulin-like growth factor 1	Stimulation of cell growth and proliferation, inhibition of apoptosis	(CA)n	4	2	Southern Indian	18-repeat of (CA) increases risk of DR	[51]
EPO	Erythropoietin	Control of erythropoiesis, stimulation of proliferation, migration and angiogenesis in hypoxic cells	rs1617640, rs507392, rs551238	7	1 and 2	Multi-ethnic, European American, Australian	TTA allele associated with PDR in European American, meta-analysis hasn´t found significant association; GCC haplotype associated with DR in Australian	[15, 52, 53]
RAGE	Advanced glycosylation end product-specific receptor	Activation of pro-inflammatory genes	rs1800624 (c. T-374A)	6	2	Indian, Chinese, African-Brazilian, Caucasian - Scandinavian	Inconsistent finding and weak association, may be interacting with glycosylated hemoglobin	[15, 54–56]
			rs1800625 (c. T-429C)	6	2	Caucasian, Indians, Danish	Inconsistent finding and weak association, functional studies show differences in polymorphic receptor activity	[55–60]
			rs2070600 (p. G82S)	6	2	Caucasian, Indian, Chinese, Malaysian	Associated with DR, no association in Malaysian	[15, 58, 61]
ACE I	Angiotensin-I converting enzyme	Component of the renin-angiotensin system—activation of angiotensin II	rs4646994 (c. G2350A)-INS/DEL at intron 16	17	1 and 2	Caucasian - Slovene, Danish; Japanese, Multi-ethnic, Iranian, Japanese, Chinese, Pakistani	D allele possibly associated with DR in T2DM in Chinese, but inconsistent finding and weak association in other populations, associated with NPDR in Pakistani	[62–66]
GSTT1	Glutathione S-transferase T1	Detoxifying enzyme—conjugation of reduced glutathione to a compounds	Null genotype	22	2	Caucasian - Slovenian	Greater risk of DR	[67]
GSTM1	Glutathione S-transferase M1	Detoxifying enzyme—conjugation of reduced glutathione to a compounds	Null genotype	1	2	Caucasian - Slovenian	Lower risk of DR	[67]
SOD2/MnSOD	Mitochondrial manganese superoxide dismutase	Decrease of ROS production (transformation to to peroxide and oxide)	rs4880 (c. C47T, p. A16V)	6	1 and 2	Slovene (Caucasian), Finnish, Indian	C allele reduces risk of DR, not confirmed in Indian population	[15, 36, 56, 65, 68, 69]
eNOS3	Endothelial nitric oxide synthases	Synthesis of nitric oxide (vasodilatation)	rs3138808 (27 VNTR intron 4 a/b)	7	2	Indians, West African, Caucasian - Brazilian	4a allele protective effect against DR	[15, 37, 70–73]
			rs1799983 (c. C894T)	7	2	Caucasian—Brazilian, Danish, Multi-ethnic	G allele increases risk but weak association	[15, 37, 50, 60, 72, 74]
			rs41322052 (c. T-784C)	7	1&2	Caucasian—Brazilian, Multi-ethnic	Inconsistent finding and weak association	[37, 71, 72, 75]
			rs2297518 (c. G-954C)	7	2	Caucasian	Protective factor against NPDR	[48]
RXRA	Retinoid X receptor alpha	Nuclear receptor—retinoic acid-mediated gene activation (antoxidants properties)	rs3132300	9	1	African American	Associated with progression of DR	[76, 77]
RXRG	Retinoid X receptor gamma	nuclear receptor—retinoic acid-mediated gene (antiproliferative effects)	rs3818569	9		Taiwanese	G allele associated with development of DR	[78]
UCP1	Uncoupling protein-1	Mitochondrial anion carrier protein (thermogenesis), protection againt oxidative stress	rs1800592 (c. A-3826G)	4	1 and 2	Brazilian, Chinese, Danish	G allele associated with increased risk of PDR	[60, 75, 79]
UCP2	Uncoupling protein-2	Mitochondrial anion carrier protein (thermogenesis), control of ROS production	rs660339 (p. A55V, 45 bp INS/DEL)	11	1 and 2	Brazilian	Risk factor for PDR	[77, 80]
TLR4	Toll-like receptor 4	Pathogen recognition and activation of innate immunity	rs10759931, rs1927914	9	2	Indian, Chinese	A,T alleles positively modulate the risk of DR, rs1927914 associated with susceptibility to DR in a Han Chinese population	[3, 55]
			rs4986790, rs4986791 (p. D299G)	9	2	Polish	G allele associated with early onset of DR	[81]
CFH	Complement factor H	Regulator of complement activation	rs800292 (p. I62V)	1	2	Chinese	Associated with DR	[82]
CFB	Complement factor B	Regulator of complement activation	rs1048709	6	2	Chinese	Associated with DR	[82, 83]
MCP-1/CCL2	Monocyte chemoattractant protein-1	Cytokine—activation of monocytes, macrophages and lymphocytes	rs1024611 (c. A-2518G)	17	2	Chinese, Korean, Japanese	G allele associated with susceptibility to DR and specifically PDR in Koreans	[84–86]
TGF-β1	Transforming growth factor-beta 1	Control of cell growth, proliferation, differentiation and apoptosis	c. T869C (p. L10P)	19	2	Multi-ethnic	Potential protect factor against DR	[15, 83]
			c. G915C (p. R25P)	19	2	Slovak	Strong risk factor for PDR	[87]
ICAM1	Intercellular adhesion molecule 1	Stabilization of cell–cell interactions and facilitation of leukocyte endothelial transmigration	rs13306430	19	2	Multi-ethnic	G allele confers protection	[15, 77]
			rs5498 (p. K469E)	19	2	Chinese, Indian, Japanese, Caucasian - Slovene	Inconsistent finding, discrepancy maybe caused by ethnicities	[55, 88–93]
SLC2A1/GLUT1	Solute carrier family 2, member 1	Transport of glucose across the plasma membranes	rs841846 (c. A26177G)	1	1 and 2	African American, Malaysian	Significant associations with severe DR, associated with progression of DR, not confirmed in Malaysian	[77, 94]
			rs841853	1	1	Malaysian, Multi-ethnic	Weak association	[77, 94]
SLC2A11	Solute carrier family 2, member 11	Transport of glucose across the plasma membranes	rs4822441	22	1	African American	Associated with progression of DR	[77]
SLC24A3	Solute carrier family 24, member 3	Sodium-calcium exchanger	rs2294895	20	1	African American	Associated with progression of DR	[77]
PPARγ	Peroxisome proliferator-activated receptor γ	Nuclear receptor—regulation of fatty acid storage and glucose metabolism, role in vascular permeability, inflammation, angiogenesis, neovascularization, and insulin resistance	rs1801282 (c. C34G, p. P12A)	3	1 and 2	Caucasian - Poland; Chinese, Danish, Multi-ethnic	G allele confers protection against DR in Caucasian but finding inconsistent - protective effect against only PDR during T2DM in Pakistan population, not for Asian patients	[60, 79, 95, 96]
			rs10510419	3	1	African American	Associated with progression of DR	[15, 95]
TCF7L2/TCF4	Transcription factor 7-like 2	Transcription factor for several genes (Wnt signaling pathway), vascular development	rs7903146, rs7901695, rs12255372	10	2	Caucasian—Italian, Chinese, Multi-ethnic	Associated with DR, cardiovascular disease and coronary artery disease, rs7903146 associated with DR risk in Caucasian	[15, 79, 96–98]
OPG/OCIF	Osteoprotegerin/osteoclastogenesis inhibitory factor	Cytokine receptor	rs2073618, rs3134069	8	2	Caucasian - Slovenian	CA haplotype increase risk of DR	[99]
PAI-1	Plasminogen activator inhibitor-1	Serine protease inhibitor—inhibitor of plasminogen activation, tissue repair and remodeling	rs1799768 (4G/5G INS/DEL)	7	2	Indian, Caucasian, Euro-Brazilian, Multi-ethnic, Pakistani	4G/5G allele increases risk but finding inconsistent, ethnicity discrepancies	[28, 55, 66, 100, 101]
MMP-2	Matrix metalloproteinase-2	Breakdown of extracellular matrix	c. C-1306T	16	2	Chinese	T allele associated with PDR	[7]
ANGPT1	Angiopoietin 1	Vascular development and angiogenesis	rs1283649	8	1	African American	Significant associations with severe DR	[77]
APOE	Apolipoprotein E	Transportation of lipoproteins, fat-soluble vitamins, and cholesterol	E2/E3/E4	19	1 and 2	Mexicans, Multi-ethnic	Inconsistent finding and weak association	[102, 103]
BBS2	Bardet-Biedl syndrome 2 protein	Unknown function and link to DR	rs4784675	16	1	African American	Significant associations with severe DR	[15]
CPVL/CHN2	Carboxypeptidase, vitellogenic-like; chimerin 2	Carboxypeptidase—unknown function/regulation of a cell growth, proliferation, and migration	rs39059	7	2	Chinese	Increases risk of DR, significant in meta-analysis	[77, 104]
			rs1002630	7	2	Taiwanese	Associated with DR and NPDR	[142]
CTSH	Cathepsin H	Lysosomal cysteine proteinase - degradation of lysosomal proteins, putative role in microcirculation changes	rs3825932	15	1	Danish	T allele associated with reduced risk of progression to PDR	[60]
DRD2	Dopamine receptor D2	Dopamine receptor—regulation of vasodilatation, aldosterone production and insulin secretion	rs7131056	11	1	African American	Significant associations with severe DR	[77]
EDN1	Endothelin-1	Vasoconstriction	rs5370 (p. K198N)	6	2	Chinese	Reduced risk in Chinese	[77, 105]
ENPP1	Ectonucleotide pyrophosphatase/phosphodiesterase 1	Insulin resistance, interaction with integrins	rs1409181	6	1	African American	Significant associations with severe DR	[77]
ERBB3/HER3	Human epidermal growth factor receptor 3	Protein-tyrosine kinase—activation of downstream signaling pathways, unknown link to DR	rs2292239	12	1	Danish	T allele associated with reduced risk of progression to PDR	[60]
FLT1/VEGFR1	FMS-like tyrosine kinase 1/vascular endothelial growth factor receptor 1	Protein-tyrosine kinase—control of cell proliferation and differentiation	rs622227	13	1	African American	Associated with progression of DR	[77]
FRMD3	FERM domain containing 3	Maintaining cellular shape, putative TSG, unknown link to DR	rs10868025	9	2	Chinese	Weak association with DR	[104]
HLA-B	Major histocompatibility complex, class I, B	Regulation of the immune system—presenting peptides on the cell surface	rs2523608	6	1	African American	Significant associations with severe DR, associated with progression of DR	[77]
HTR1B	Serotonin receptor 1B	GPCR for serotonin—regulation of the serotonin, dopamine, and acetylcholine release, putative regulator of retinal blood flow	rs1228814	6	1	African American	Significant associations with severe DR	[77]
HTRA1/ARMS2	HtrA serine peptidase 1/age-related maculopathy susceptibility 2	Serine protease—regulation of insulin-like growth factors, putative regulator of cell growth and neovascularization	rs11200638, rs10490924	10	2	Indian	Marginal association with DR	[55]
IL-10	Interleukin-10	Cytokine—pleiotropic effects in immunoregulation and inflammation	n. A-1082G	1	2	Indian	G allele is risk factor for PDR	[113]
INSR	Insulin receptor	Activation of the insulin signaling pathway	rs10500204	19	1	African American	Associated with progression of DR	[77]
ITGA2B1	Integrin α2β1	Cell–cell and cell-extracellular matrix interactions	RFLP - Bgl II	5/10	2	Japanese, Caucasian	Risk factor for DR	[15, 106, 143]
ITGB5	Integrin β5	Cell–cell and cell-extracellular matrix interactions	rs9865359	3	1	African American	Associated with progression of DR	[15]
MTHFR	Methylenetetrahydrofolate reductase	Remethylation of homocysteine to methionine	rs1801133 (c. C677T)	1	2	Japanese, Euro-Brazilian, Multi-ethnic, Turkish	Controversial findings, T allele possible increases risk of DR because of hyperhomocysteinemia	[28, 77, 107, 108]
NPY	Neuropeptide Y (p. L7P)	Vasoconstriction, angiogenesis	rs16139	7	2	Finnish	C allele increases risk but weak association	[144]
OLR1	Oxidited low-density lipoprotein (lectin-like) receptor 1	Recognition, internalization and degradation of oxidized low-density lipoprotein, putative regulator of Fas-induced apoptosis	rs2742115	12	1	African American	Associated with progression of DR	[77]
PEDF/SERPINF1	Pigment epithelium derived factor/serpin peptidase inhibitor, clade F member 1 (alpha-2 antiplasmin)	Antioxidative properties, inhibition of angiogenesis, neurotrophic factor (neuronal differentiation in retinoblastoma cells)	rs12150053, rs12948385, rs8697961, rs1126287	17	2	Multi-ethnic	Not associated with DR	[8, 15]
PON1	Paraoxonase 1	Cellular antioxidant—inhibition of HDL oxidation	rs662 (p. Q192R)	7	2	Multi-ethnic	Inconsistent finding and weak association	[15, 109]
			rs854560 (p. L55M)	7	1 and 2	Multi-ethnic	Associated with DR	[109]
PON2	Paraoxonase 2	Cellular antioxidant, hydrolytic activity—a putative role in defense responses to pathogenic bacteria	rs7493 (p. S311C)	7	1 and 2	Multi-ethnic	Inconsistent and weak association	[77]
			s12026 (p. A148G)	7	1 and 2	Multi-ethnic	Inconsistent and weak association	[109]
PROS1	Protein S	Cofactor for the anticoagulant protease	rs13062355	3	1	African American	Significant associations with severe DR	[77]
PSMD9	Proteasome 6S subunit, non-ATPase, 9	Part of multicatalytic proteinase complex (proteasome)	rs74421874, rs14259, rs3825172	12	2	Italian	Associated with DR	[110]
ROBO2	Roundabout, axon guidance receptor, homologue 2	Axon guidance and cell migration, unknown link to DR	rs10865559	3	1	African American	Significant associations with severe DR	[77]
ROCK2	Rho-associated, coiled-coil containing protein kinase 2	Serine/threonine kinase—regulation of cytokinesis, smooth muscle contraction, the formation of actin stress fibers and focal adhesions, and the activation of the c-fos serum response element	p. T431N, p. R83K	2	1 and 2	Turkish	No association	[15, 111]
Romo-1	Reactive oxygen species modulator 1	Mitochondrial membrane protein—increase of the level of reactive oxygen species in cells	rs6060566	20	2	Caucasian	Independent risk factor for DR	[112]
TF	Transferrin	Transportation iron from the intestine, reticuloendothelial system, and liver parenchymal cells to all proliferating cells in the body	rs3811647	3	1		Associated with progression of DR	[77]
TNF-α	Tumor necrosis factor-alfa	Multifunctional proinflammatory cytokine (cell proliferation, differentiation, apoptosis, lipid metabolism, and coagulation)	rs361525 (c. G-238A), rs1800629 (c. G-308A), rs1799724 (c. C-857T)	6	2	Indian, Caucasian - Brazilians	AA genotype of rs361525 confers risk for pathogenesis of PDR in Indian, rs1800629 associated with PDR in Caucasian - Brazilians	[113–115]
TNF-β/LTA	Tumor necrosis factor-beta (lymphotoxin-alpha)	Cytokine— inflammatory, immunostimulatory, and antiviral responses, the formation of secondary lymphoid organs, apoptosis	NcoI	6	2	Caucasian - Slovak	β2 allele is genetic factor for incidence of PDR in T2DM	[15, 116]
			(GT)n microsatellite	6	2	Asian Indian	Allele 4 (103 bp) is a low risk for developing retinopathy, allele 8 (111 bp) is associated with PDR	[114, 115]
VDR	Vitamin D receptor	Nuclear hormone receptor for vitamin D3, associated with insulin secretion and sensitivity, anti-proliferative and anti-angiogenic effect, regulator of apoptoses	rs10735810	12	1 and 2	Multi-ethnic	T allele increases risk but weak association	[15]
			rs2228570	12	2	Han Chinese	T allele increases risk of DR onset	[117]
			rs1544410	12	2	Polish, Korean	Protective effect against DR in Korean	[118, 119]

*Ch*. chromoseme, *Ref*. references, *Multi-ethnic* findings of studies regardless of ethnicity or from meta-analyses, *TSG* tumor suppressor gene, *GPCR* G protein-coupled receptor

The mitochondrial aldehyde dehydrogenase 2 (ALDH2), expressed in vasculature, detoxifies reactive aldehydes formed from glucose and lipids, also prevents creation of AGE (advanced glycation end products) [32]. Morita et al. have reported a substantial relation between the ALDH2*2 allele and the incidence of DR in their study.

## Growth factors with role in DR

Vascular endothelial growth factor (VEGF) is one of the major factors in angiogenesis and influences vascular permeability of endothelial cells. VEGF is activated by microvascular changes induced by hypoxia during DM and also by hyperglycaemia [120]. Activation of VEGF leads to the destruction of the blood retinal barrier (BRB), the development of diabetic macular oedema and neovascularization typical for PDR. At the same time, elevated serum and vitreous levels of VEGF have also been described in eyes of patients with PDR [121]. Anti-VEGF therapies have led to the improvement of the patients´ condition and to the deceleration of retinal vessels proliferation [122]. Studies have revealed several polymorphisms in the *VEGF* promoter (rs2010963, rs25648, rs1570360, rs3095039, rs35569394, rs699947, rs13207351, rs735286, rs2146323, rs833061, rs302502, rs10434, rs833068 and rs833070) with possible associations with DR [15, 33, 42, 44]. Rs2010963 (−634C/G) has been associated with DR in Japanese and Indian populations [34, 35] whereas G allele of rs2010963 has significant protective effect against NPDR in patients with T2DM. Rs2010963 is also associated with higher risk of macular oedema in Japanese population [123]. There are constantly emerging studies identifying new polymorphisms in *VEGF* gene with possible connections to DR which underlines importance of this gene in the development of DR.

Other growth factors with a possible function in the pathology of DR are the basic fibroblast growth factor (bFGF) and insulin-like growth factor 1 (IGF-1). The bFGF is important for tissue repair and is angiogenic factor. Studies have revealed increased level of bFGF in patients with PDR and it seems to stimulate VEGF production. IGF-1 regulates the proliferation and differentiation of several cell types. Levels of intravitreal IGF-1 were found to be significantly increased in the eyes of patients with PDR compared to those of controls [124]. Variants identified to date are summarized in the Table 1.

Erythropoietin (EPO) plays an important role in stimulation of bone marrow stem cells, erythropoiesis, proliferation, migration, and angiogenesis in hypoxic vascular endothelial cellsStudy has reported a elevated concentration of EPO in the vitreous of DM and PDR patients compared to controls [52]. There are two studies which have reported the association of rs1617640, rs507392, and rs551238 with the development of DR, but these studies report different findings. Tong et al. have determined the TTA haplotype as a risk contributor in European American population, whereas Abhary et al. have associated the GCC haplotype with DR in Australian population [52, 53].

**Fig. 3:**
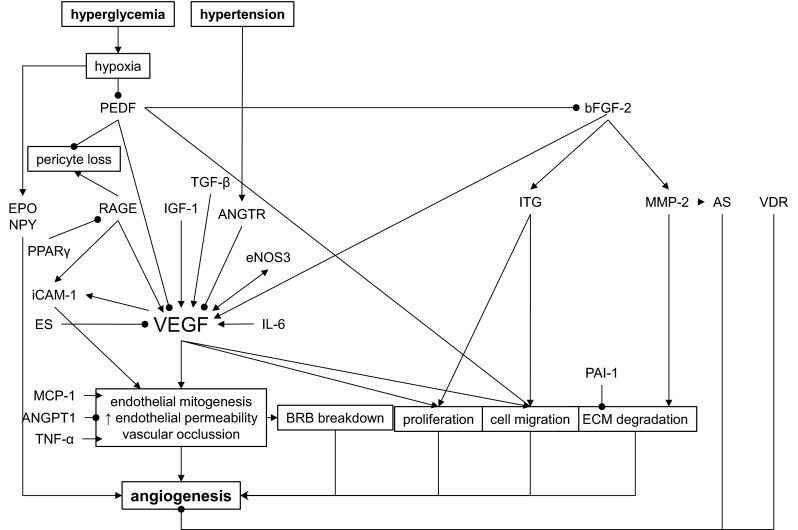
Genes harboring DNA polymorphisms involved in angiogenesis during diabetic retinopathy (DR). *AS* angiostatin, *ES* endostatin, *BRB* blood retinal barrier, *ECM* extracellular matrix, • inhibition

Interaction of various growth factors, cytokines, cell signalling molecules and extracellular matrix are essential for angiogenesis during DR [125] while VEGF plays crucial part [126] (Fig. 3).

## Receptor for advanced glycation end products and cytokines

Hyperglycaemia causes nonenzymatic glycation of proteins and lipids and the creation of AGE. Accumulation of AGE leads to tissue damage by the formation of a covalent crosslinks between proteins, which alter structure and function of proteins. Another feature of AGE is its ability to interact with different surface receptors, such as the receptor for advanced glycation end products (RAGE). RAGE is a immunoglobulin and its activation leads to cytokine secretion. Cytokines accelerate the advance of diabetic complications by supporting proinflammatory processes and increasing endothelial permeability [127]. AGEs are found in the retinal vessels of diabetic patients where their levels correlate with those in the serum as well as with severity of retinopathy [128]. The c. T–374A (rs1800624), p. Gly82Ser (rs2070600) and c. T-429C (rs1800625) polymorphisms in the RAGE gene are associated with DR in Caucasians and Asian Indians [54, 57, 129, 130], but the association was not confirmed in Chinese [131].

## Dysregulation of RAAS system

The rennin-angiotensin-aldosterone system (RAAS) is an endocrine system involved in the regulation of blood pressure and fluid balance. Patients with diabetes show dysregulation of RAAS system, namely angiotensin converting enzymes I and II (ACEI, ACEII) and angiotensin receptors which are upregulated in retina during PDR independently of blood pressure [132]. ACE converts angiotensin I (ATI) to angiotensin II (ATII) which mediates its haemodynamic effects through the angiotensin receptor ANGTR1 and ANGTR2. ATII in the eye regulates promotion of capillary growth, cell growth, intraocular blood flow and pressure,, enhances vascular permeability, increases oxidative stress and via the expression of several growth factors including VEGF, IGF-1 and PDGF [15]. ACE inhibitors, angiotensin receptor blockers prevents neovascularization, reduce the incidence and progression of DR in T1DM. Studies have proposed that *ACEII* is also involved in PKC activation [133]. Meta-analysis suggested that *ACE* I/D polymorphism (insertion/deletion of a 287 bp Alu sequence in intron 16) may be associated with PDR [62].

## The other polymorphisms modulating risk of DR

The retina is very sensitive to damage by oxidative stress. Oxidative stress is strongly implicated in the pathogenesis of DR, therefore the role of detoxifying enzymes, such as glutathione S-transferases (GST), was considered in the development of DR. Studies have shown that *GSTT1*-null genotype is found more frequently in the cases with DR in Caucasians patients with T2DM compared to controls, so the *GSTT1*-null genotype can be a risk factor for DR The individuals homozygous for the *GSTT1*-null allele had more generalized vasculopathy that leads to increased risk of sight threatening DR. In contrast, the *GSTM1*-null genotype may confer protection against development of DR in people with T2DM [67], but at the same time this polymorphism confers elevated risk for lung cancer [134]. There are reports that deficiency in GSTM1 leads to slower excretion of isothiocyanates. Isothiocyanates also suppress expression of VEGF which is the main inductor of retinal neovascularization in diabetes [67].

Oxidative stress induces a large amount of ROS and is assumed to damage the mitochondrial DNA. Mitochondrial manganese superoxide dismutase (*MnSOD*) prevents an excessive production of ROS by dismutation of superoxide radicals into hydrogen peroxide and hence defends the retinal endothelial cells from oxidative damage. The polymorphism rs4880 (c. C47T, p. A16V) affects a mitochondrial processing efficiency under oxidative stress and has been associated with DR in some studies [36, 68, 69].

Regarding PDR, presence of the 4a/4a genotype of the VNTR polymorphism for endothelial nitric oxide synthase (*eNOS*) has been associated with 3.4 times increased risk of PDR in Caucasian patients with T2DM [135]. In contrast, other studies have proposed that the 4a allele has a protective effect against DR [70, 71]. NO synthesized by eNOS is an endogenous vasodilator and has a role in induction of angiogenesis and regulation of VEGF expression. NO levels are significantly elevated in PDR patients relative to nondiabetic subject.

A study in 2008 revealed that retinoid-X receptor alpha (RXRA) possessess antioxidants properties and is associated with the development of DR [76]. Polymorphism rs3132300 has been linked with a progression of DR in T2DM in African American population. Also, polymorphism rs3818569 of the retinoid-X receptor gamma (*RXRG*) has been found to be connected with an increased DR risk in the Taiwanese population [78].

Uncoupling protein 1 (UCP 1) is the mitochondrial inner membrane electron carrier that has a part in protection against oxidative stress. It has been proposed that *UCP 1* and its product play role in insulin resistance when oxidative stress pathways are activated. SNP rs1800592, which is located in the promoter of the *UCP1*, has been shown to be associated with glucose homeostasis, adiposity and obesity, as well as changes in the body mass index (BMI) and body weight, resulting from metabolic disorders. UCP 1 has been implicated as a candidate marker for a risk factor of DR and the rs1800592 (c. A-3826G) polymorphism has been associated with PDR [75, 79]. Uncoupling protein 2 (UCP2) regulates production of reactive oxygen species (ROS) by mitochondria. Overproduction of ROS is associated with diabetic retinopathy (DR), thereby UCP2 gene polymorphisms can be involved in the development of this complication. rs660339 can be a relevant risk factor associated with PDR in both type 2 and 1 of diabetes [77, 80].

The inflammatory processes are a major part of the DR pathophysiology. They are often regulated by inadequate activation of members of the immune system. Toll-like receptor 4 (TLR4) takes part in the activation of a pro-inflammatory response by the ligand-depended activation of the nuclear factor-κB (NF-κB) pathway. Any deregulation of *TLR4* signaling due to single nucleotide polymorphisms (SNPs) in the extracellular domain of *TLR4* may alter the ligand binding capacity and hence disturb the balance of pro- and anti-inflammatory cytokines [81]. It has been reported that rs4986790, rs4986791, rs10759931 and rs1927914 in TLR4 positively modulate the risk of DR [3, 81, 136].

There is increasing evidence from in vitro and in vivo studies that suggests a pathogenic role of the complement system in the development of diabetic angiopathy. In these studies, increased expression of several complement factors, namely, complement factor H (CFH), complement factor B (CFB), component 3 (C3), and component 5 (C5), has been observed in the vitreous of DR patients. CFH and CFB (an antagonist of CFH) contribute to the regulation of the activation of complement cascade. Polymorphism rs800292 (p.I62V) in *CFH* affects protein-binding affinity with C3b and subsequently activation of the complement alternative pathway. A synergy effect between *CFH* rs800292 and *CFB* rs1048709 conferring a significantly increased risk for DR has been identified in the study of Wang [82].

Studies have reported significantly increased levels of monocyte chemotactic protein 1 (MCP-1) in aqueous and vitreous conditions in DR patients. MCP-1 has an ability to activate monocytes, macrophages and lymphocytes. Hyperglycaemia accelerates MCP-1 production in vascular endothelial cells and retinal epithelial cells which can lead to neovascularization and increased permeability of retinal vessels typical for PDR. Moreover rs1024611 polymorphism has been associated with DR in the Japanese, Korean, and Chinese populations [84–86].

Transforming growth factor β1 (*TGF-β1*) has an important role in angiogenesis, endothelial cell proliferation, adhesion and the deposition of extracellular matrix. The *TGF-β1* gene may be involved in the development of DR through induction of angiogenesis and BRB breakdown. C. T869C (p. L10P) polymorphism has been associated with a protective effect against DR [15, 83].

Intercellular adhesion molecule-1 (*ICAM-1*) has a major role in mediating the adhesion of circulating leukocytes to the blood vessel wall and transendothelial migration to the vascular intima. The increased retinal expression of *ICAM-1* is thought to play a key role in leukostasis-mediated BRB breakdown, capillary occlusion and endothelial cell damage in DR [88]. Polymorphisms of *ICAM-1* gene might have a role in modulation of its own gene expression but findings about p. K469E polymorphism (rs5498) are inconsistent across multiple studies [55, 88–91]. The G allele of rs13306430 could confer protection against DR in T2DM patients [15].

Solute carrier family 2(*SLC2A1)*, also known as facilitated glucose transporter (*GLUT1*) is expressed in endothelial cells of the BRB where SLC2A1 is the prevalent glucose transporter. Functional loss of BRB is typical for DR and studies have shown that patients with DR have high expression of *GLUT1* in endothelial cells. *SLC2A1* c. A26177G polymorphism has been associated with DR in a study of Ng [94].

The role of peroxisome proliferator-activated receptor γ (*PPARγ*) in DR pathogenesis has come to forefront mainly because of the protein’s role in vascular permeability, inflammation, angiogenesis, neovascularization, and insulin resistance, all of which contribute to the onset and severity of DR. However, the studies describing associations of *PPARγ* polymorphisms and DR have been inconsistent [15, 95].

Transcription factor 7-like 2 (*TCF7L2*/*TCF4*) is a key component in the regulation of fundamental processes such as vascular development. It has been found to mediate pathological neovascularization in PDR. Common variant rs7903146 in *TCF7L2* has been reported to be strongly associated with T2DM and also with PDR in Caucasian [96]. An Italian study observed associations between *TCF7L2* variants (rs7903146, rs7901695 and rs12255372) and DR, cardiovascular disease and coronary artery disease [97].

Osteoprotegerin (OPG), also called the osteoclastogenesis inhibitory factor (OCIF), is an important regulatory molecule in the vasculature. Rs2073618, rs3134069 polymorphisms have been linked with DR [99].

Plasminogen activator inhibitor-1 (*PAI-1*) is an inhibitor of plasminogen activation and is involved in tissue repair and remodeling. PAI-1 plays a crucial part in the regulation of intravascular fibrinolysis which is part of DR pathophysiology. Studies have investigated the connection between *PAI-1* 4G/5G and DR risk but findings have been inconsistent, maybe due to ethnicity discrepancies [15, 100, 101].

Matrix metalloproteinases (MMPs) are proteolytic enzymes that degrade extracellular matrix (ECM) components. MMPs also regulate cell proliferation, neovasculogenesis and tissue remodelling because degradation of the extracellular matrix (ECM) proteins of the basement membrane is necessary for endothelial cells to migrate, proliferate, and to form capillaries. Increased expression of MMP-2 may expedite degradation of the type IV collagen and the gap junction protein, accelerating the vascular complications of diabetes. It has been reported that c. C-1306T polymorphism seems to be genetic susceptibility factor for the development of DR [7].

In this review, we discussed candidate genes and polymorphisms with the highest genetic association with DR, or those most frequently analysed in studies in different population. The other genes and their polymorphisms associated with DR are summarized in Table 1 in alphabetical order. Studies concerning these genes have reported very weak or borderline associations, had small sample sizes and most of them have failed replication in other populations [137]. We did not include studies and polymorphisms that have been done on only one population and showed no associations with DR. None of the polymorphisms identified by candidate gene studies have achieved widespread acceptance as a marker of high risk of diabetic retinopathy. In part, this may be because of the complexity of DR which probably has more multifactorial, polygenic and environmental contributors to its pathophysiology.

In our review, we have included a number of genes in which multiple alleles or nearby SNPs have varying strengths of association or significance in relation to diabetic retinopathy. Possible explanation for this is variable association between the SNPs themselves with the causative change depended also on populations.

Another one of the possible reasons for the setback of the gene candidate approach is focusing on single SNPs when the linkage disequilibrium does not have to manifest [16]. It would be potentially useful to focus on haplotypes associated with DR instead of solely on SNPs. In addition, insufficient sample sizes to detect the modest effect of polymorphisms, incomplete coverage of variation in candidate genes and incorrect hypotheses about genes in the pathophysiology of DR are some of the reasons for controversial success of candidate genes studies. Based on these weaknesses of standard candidate gene studies, two recent studies have examined a higher number of candidate genes for DR in sample sizes larger than those used previously, in an approach that mimics a genome-wide approach [77, 138]. The first study, which examined 193 candidate genes with DR of type 1 diabetic African-Americans, found genetic associations in 13 genes with progression of DR and the polymorphisms are listed in Table 1 [77]. Identified genes are involved in pathways related to glucose metabolism, inflammatory processes, angiogenesis/vascular permeability, insulin signalling, retinal development, or blood pressure regulation. The second study, the Candidate gene Association Resource (CARe) has not confirmed connection between previously associated gene from numerous previous independent studies and DR [138]. The most interesting findings from this study are the variants in the P-selectin (*SELP*) (rs6128) and in the iduronidase (rs6856425) that have shown to be significantly linked with DR in European Americans, but were not seen in African-Americans, Hispanic Americans, or Asian Americans.

It is possible that the potential success of candidate gene studies lies in better characterization and definition of clinical phenotypes of DR, represented by specific patterns of severity and progression of DR. Only then, studies of candidate genes are worth pursuing, involving appropriately well-defined subgroups of patients [139]. Furthermore, it would be worthwhile to expand studies to those genes mutations in which are known to initiate hyperglycaemia [140] and indirectly lead to development of DM and DR.

## Conclusion

DR remains to be one of the most complex, heterogeneous, multifactorial disorders in any genetic studies. It is one of the leading causes of blindness and visual impairment in the world and treatments options are limited. Research worldwide is focused on understanding the pathogenic mechanisms in DR with the key goal to prevent this disease and developing new drugs for treatments. There is a common consensus that the susceptibility to DR is contingent to a great amount of relatively common allelic variants with a modest effect, and how these genes interact among themselves and with environmental influences. Each of the allelic variants increased risk of DR by a small portion in overall susceptibility. The identification of genetic susceptibility loci for DR by genetic studies has not proved notably successful thus far, given the often contradictory and inconclusive results. It is obvious that the study of the DR genetics is still poorly developed and stands against numerous challenges. The most common approaches in studying this complex disease are insufficient for elucidating pathology of DR. At the same time, most studies of new possible treatments disregarded genetic background of the patietns, which could contribute to the treatment setback. Possible new NextGen sequencing methods and approaches based on interconnection of various omics, such as genomics, especially pharmacogenetics, transcriptomics, proteomics and metabolomics, will bring new breakthrough findings in the future. At this time, we can surely state that there is a long way ahead to fully understand this complex disease.
